# Gene Expression Signatures of Gliovascular Cells Associated with Microinfarct Burden

**DOI:** 10.1002/alz70861_108464

**Published:** 2025-12-23

**Authors:** Kyra M Clarke, William Cole Corbett, Angelique D. Gonzalez, Ali Ghaseminejad‐Bandpey, Matthew B Dopler, Mohammad Alhneif, Julie Parker‐Garza, Benjamin Danner, Sahana Babu, Oluwaseun Ogunbona, Shahroo Etemadmoghadam, Lon R White, Habil Zare, Margaret E Flanagan

**Affiliations:** ^1^ Glenn Biggs Institute for Alzheimer’s & Neurodegenerative Diseases, UT Health San Antonio, San Antonio, TX USA; ^2^ Glenn Biggs Institute for Alzheimer's & Neurodegenerative Diseases, UT Health San Antonio, San Antonio, TX USA; ^3^ Department of Pathology and Laboratory Medicine, UT Health San Antonio, San Antonio, TX USA; ^4^ Pacific Health Research and Education Institute, Honolulu, HI USA; ^5^ Glenn Biggs Institute for Alzheimer’s & Neurodegenerative Disease, UT Health San Antonio, San Antonio, TX USA; ^6^ Department of Cell Systems & Anatomy, UT Health San Antonio, San Antonio, TX USA

## Abstract

**Background:**

Cerebral small vessel disease (cSVD) is a major contributor to vascular cognitive impairment and is increasingly recognized as a significant co‐pathology in Alzheimer’s disease and related dementias (ADRD). Cortical microinfarcts‐ microscopic ischemic lesions that are frequently missed on standard neuroimaging‐ are lesions indicative of underlying cSVD and prevalent among patients with dementia. Understanding the molecular and cellular consequences of microinfarcts is essential to elucidating how vascular injury interacts with neurodegenerative processes in ADRD.

**Method:**

We utilized the NanoString GeoMx DSP platform to profile gene expression in postmortem human brain tissue from four subjects, stratified by microinfarct burden and matched for arteriosclerosis and AD Neuropathologic Change severity. These samples were confirmed not to have microinfarcts in the regions examined to investigate diffuse changes due to global cerebral hypoxia vs. lesion specific alterations. Areas of interest (AOIs) were selected from the middle frontal gyrus and basal ganglia for a total of 190 AOIs (54 – 929 cells per AOI; median = 287). Whole transcriptome sequencing was performed on spatially segmented astrocytes, microglia, and endothelial cells.

**Result:**

Differential gene expression and pathway overrepresentation analysis revealed cell and region‐specific transcriptional changes in response to increasing microinfarct burden. Strikingly, the greatest transcriptional changes in gliovascular cells were observed at early stages of pathology. These findings suggest that early gliovascular changes may represent compensatory responses or the onset of pathological dysfunction driven by cSVD.

**Conclusion:**

Increasing microinfarct burden differentially impacts gene expression across brain regions and cell types. Spatial transcriptomics can help identify molecular signatures and candidate biomarkers of cSVD. These findings could ultimately contribute to improved detection of microvascular pathology and a better understanding of its role in AD and related dementias.

